# Do political protests mobilize voters? Evidence from the Black Lives Matter protests

**DOI:** 10.1007/s11127-022-00998-y

**Published:** 2022-09-26

**Authors:** Oliver Engist, Felix Schafmeister

**Affiliations:** Department of Economics, Stockholm School of Economics, Stockholm, Sweden

**Keywords:** Political protests, Voting, Black Lives Matter, Voter registration, Election, D72, D74

## Abstract

In this article, we study the local political mobilization effects of political protests in the context of the Black Lives Matter (BLM) movement. We analyze monthly voter registration data from 2136 US counties across 32 states, leveraging variation in the exposure to BLM protests across counties in a two-way fixed-effects framework with a matched control group. In contrast to previous studies, which reported substantial mobilization effects of local protests in other contexts, we show that voter registrations in the aggregate were insensitive to the presence of local BLM protests. We further disentangle the effects along party lines and the degree to which protests were associated with violent behaviors and find similarly insignificant effects. We present some preliminary evidence that the large scale of the protests and their extensive news coverage might have reduced the importance of experiencing a protest firsthand.

## Introduction

Many powerful political movements arise from seemingly insignificant events that set in motion a cascade of consequences. In some cases, the process ultimately results in a change of government or the entire dissolution of a nation. Early theoretical studies struggled to explain the emergence of rebellions, since the reward they provide is a public good, whereas the potentially large costs of participation are borne by the individual (Tullock, 1971). Subsequent literature proposed a variety of explanations consistent with rational choice theory that can reconcile this seeming paradox of revolution. These explanations include bloc mobilization (Oberschall, 1994), uncertainty about the repressive capabilities of the regime (Boix & Svolik, 2013), and social preferences (Shadmehr & Bernhardt, 2011).^1^ In a similar spirit, Kuran (1989) described a framework in which privately held and publicly voiced political preferences can diverge. This results in a bandwagon effect, where individuals hold their political views private until a sufficiently large number of individuals voice similar views.

A recent example of such a movement is Black Lives Matter (BLM), which, although officially founded in 2013, mushroomed into a global movement of an almost unparalleled scale following the death of George Floyd in police custody on May 25, 2020. Driven by concerns about perceived racial injustices, protests occurred across the United States, as well as in many cities worldwide.

Despite the large scale of the movement and its associated protests, little is known about its political consequences. Although the protests primarily targeted perceived racial injustices, they commonly involved calls to get out the vote and emphasized the importance of registering to vote to achieve political change (New York Times, 2020). Moreover, the protests received a large amount of media coverage across the political spectrum. However, this coverage was marked by a deep ideological divide, as some conservative commentators emphasized the occurrence of violent outbursts at some of these protests, seeking to reinforce their narrative that a Democratic government would threaten public safety (FiveThirtyEight, 2020).

These factors suggest that the BLM protests might have contributed in important ways to the record-breaking voter registration levels and turnout observed in the 2020 presidential election by encouraging voters in support of the movement, as well as those opposing it, to cast their vote. In this study, we focus specifically on the impact of local protests on the political mobilization of previously unregistered voters by comparing temporal patterns in voter registration across observationally similar communities with and without large-scale BLM protests.

The vast majority of US states require voters to register to vote, a procedure that has long been acknowledged as potentially detrimental to voter turnout, since it compels prospective voters to expend energy at a time when political interest is relatively low (Highton, 1997; Rosenstone & Wolfinger, 1978). Recent years have seen a variety of efforts to increase political participation, including the abolition of voter registration deadlines (Brians & Grofman, 2001), widespread registration drives (Nickerson, 2015), and automatic voter registration when a citizen engages with government entities (McGhee et al., 2021). Despite these advances, there remains a substantial population of eligible yet unregistered voters (Pew Charitable Trusts, 2012), particularly among low-income Americans (Brians & Grofman, 1999). This gap is highly relevant, since interventions aimed at increasing voter registrations have been shown to translate directly into higher voter turnout (Nickerson, 2015).

The above-cited research thus suggests that drivers of voter registrations are an important factor to study as we seek to understand political participation in the United States.

A distinct advantage of voter registration data over traditional measures of electoral participation is their availability with high frequency. Compared to biennial turnout data, this data availability considerably mitigates potential confounding. One might be concerned about the possibility that protests are endogenous to places where they maximize political mobilization due to unobserved factors, such as the potential for new registrations. As outlined by Azam (2019), such behavior would lead to a biased estimate of the effect of protests in purely cross-sectional regressions. By observing voter registrations in a panel, we can account for such unobserved factors if they are constant during the observed time period (Wooldridge, 2015). We argue that focusing on a short time horizon before and after the protests lends credibility to the assumption that confounding variables did indeed remain constant during our sampling period.

However, the use of voter registrations as an outcome also has some limitations that qualify our conclusions in important ways. First, registrations capture only the political engagement of previously unregistered voters. Although studying this population is interesting in its own right, its non-representative nature limits the extent to which findings can be extrapolated to the electorate as a whole (Jackman & Spahn, 2021). Second, the analysis of timing variation in voter registrations requires assumptions about why individuals prefer to register to vote at one point in time rather than another. While time-varying costs of registration are likely important (Cantoni, 2020; Kaplan & Yuan, 2020), we argue that the salience of political events can be a strong motivating factor, especially considering the availability of online voter registration in most states by 2020.

Our research contributes to several strands of the literature in economics and political science. Most notably, we analyze the impact of political protests on voter mobilization. This question has previously been studied by Madestam et al. (2013), who found that protests by the Tea Party movement led to a local increase in the vote share for the Republican party. We add to this body of knowledge by providing estimates on the local political mobilization effects of another large-scale political movement, using an alternative outcome and identification strategy. To the best of our knowledge, we are the first to estimate the effect of the Black Lives Matter protests on political mobilization.^2^

We further contribute to the vast literature on how voters react to dramatic external events. For example, terrorism has been found to affect voting, even though the violence was committed by independent actors without the support of political parties (Geys & Hernæs, 2020; Montalvo, 2011). The lootings and riots that accompanied some of the BLM protests provided conservative commentators with a powerful narrative contending that under a Democratic government, lootings and riots would be the norm.^3^ Alternatively, the BLM movement can be viewed as an expression of dissatisfaction with prior policy. Voters might be motivated by seeing many citizens openly demand more progress on racial equality and policing. The previous literature on retrospective voting has generally confirmed that voters hold policymakers accountable for failure to control crime (Arnold & Carnes, 2012; Bateson, 2012) or failures of the education system (Holbein, 2016). Experimental evidence has shown that the context and framing in the media matter in determining how voters attribute blame (Healy & Malhotra, 2013; Malhotra & Kuo, 2008). Since conservative and liberal leaning voters consume different news sources (Allcott & Gentzkow, 2017; Bakshy et al., 2015; Gentzkow & Shapiro, 2011), we can expect that they would receive different interpretations of the legitimacy of the Black Lives Matter protests, which might change the extent to which voters are mobilized.

Our results do not support the notion that local political protests affected voter mobilization, either in the aggregate or on either side of the political spectrum. Furthermore, we find similar null effects also for the subset of counties in which protests turned violent, although the considerably reduced sample size does not allow us to confidently rule out meaningful effect sizes.

Although our results stand in contrast to earlier findings in the literature on the mobilization effects of political protests, these differences might be attributed to the scale of the BLM movement and its extensive media coverage. Similar to the prior literature, our analysis cannot identify the overall impact of the BLM movement but, rather, focuses on the differential in the mobilization effects induced by local protests. The vast media coverage of the BLM movement might have reduced the importance of local exposure, thus contributing to the null effect we estimate in this study.^4^ The major national cable TV networks spent almost 2.5 hours per day reporting about the protests on the weekend after George Floyd’s death (FiveThirtyEight, 2020). In addition, we show that despite considerable variation in interest across states, even areas with little exposure to local protests exhibited substantial interest in BLM, as measured by Google Trends data.

Section 2 of this paper describes our data sources and presents descriptive statistics of the sample; Sect. 3 describes our empirical strategy; Sect. 4 indicates our main results, heterogeneity analyses and robustness checks; and Sect. 5 provides a brief conclusion.

## Data

### Voter registration data

With the exception of North Dakota, all US states require citizens to register to vote before they can cast a ballot in federal, state, and local elections. Even though voter registration is a nearly ubiquitous requirement, the regulations and procedures underlying the registration process differ widely across states. In 2020, about one-third of states permitted same-day voter registration, while other states required voters to register until several weeks before election day. Furthermore, states differed in the availability of online voter registration as well as systems that automatically register eligible individuals to vote when they interact with other government agencies.

Similar to the differences in regulations regarding the timing of voter registration across states, there are also considerable differences in how voter registration statistics are released to the public. Some states release regular updates of their statewide voter file, whereas others merely release infrequent reports of aggregated voter registration numbers. In an attempt to overcome these data availability issues, various data aggregators have attempted to compile comprehensive nationwide voter files by combining separate statewide voter files. Although these undertakings have created powerful resources that have recently been used in academic research (Cantoni & Pons, 2021; Hassell et al., 2020), their usage is restricted. Since our estimation strategy relies only on aggregate data, we instead opted to collect publicly available reports released by the offices of secretaries of state and of state election boards.

To nonetheless achieve a wide coverage of states, we aimed to collect monthly (or higher-frequency) reports of county-level voter registration statistics released during 2020, leading up to the general election. If no such reports were publicly available, we contacted the relevant agency and requested similar files. This approach resulted in a sample of 17,088 monthly county-level observations from 32 states spanning a period from February to September 2020.^5^ Since not all states record party affiliations, we observed voter registrations by party only for a subset of 1276 counties in 22 states.^6^ While our coverage of US states is incomplete, it is representative of the United States as a whole.^7^ Nonetheless, in an attempt to quantify the importance of this selection, in Sect. 4.1 below we show that the inclusion (or omission) of any one state does not fundamentally alter our main conclusions.

We must account for several features of the data in our analyses. First, in some states, the voter registration reports are not compiled at regular intervals, but instead are produced to reflect voter registration totals around the time of specific events. Hence, although the data frequency is roughly monthly, the precise number of days between two monthly observations can vary. Second, though most states compile their voter registration reports at the beginning or end of each month, a small subset of states reports voter registration totals in the middle of the month. Third, since most states report voter registration totals rather than new registrations, the numbers are affected by voter list purges, resulting in occurrences of negative monthly changes. We account for these issues by including state-by-period fixed effects in all our analyses, thereby effectively comparing counties within the same state in a given time period.

**Fig. 1:**
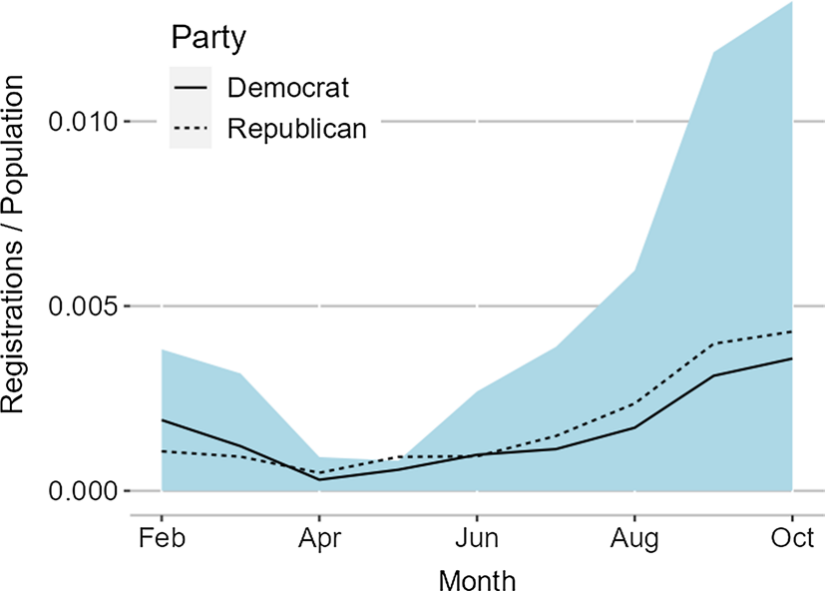
Monthly registrations per capita. *Notes:* The blue shaded area shows new registrations during a specific month across all states and party affiliations in our sample. The lines show the number of new registrations by party affiliation, relative to the population of the states in which party affiliation is available. Since party affiliation is available only for a subset of the states, the lines do not have to add up to the blue shaded area. Reporting dates vary across states; therefore, observations were assigned to the closest end of month (e.g., voter registrations for June might be recorded on June 25 in one state and July 3 in another). (Color figure online)

Figure 1 shows the evolution of voter registrations over our sample period. Since the sample coverage differs toward the end of the sample period, registrations have been standardized by the population in the sample to make the numbers comparable. Following a period of very low registration numbers in April and May, the number of monthly registrations picked up considerably in June, both in the aggregate and for each party. Although this increase roughly coincides with the height of the BLM protests, the aggregate data do not allow us to distinguish whether the increase was caused by the BLM protests or other factors such as primary elections or approaching registration deadlines.

### Protest data

The data on protests were compiled by the Armed Conflict Location and Event Data Project (ACLED; https://acleddata.com/special-projects/us-crisis-monitor/) and retrieved in December 2020. The dataset contains all events of political violence, demonstrations, or strategic developments in the United States between May 1 and December 12.

**Fig. 2:**
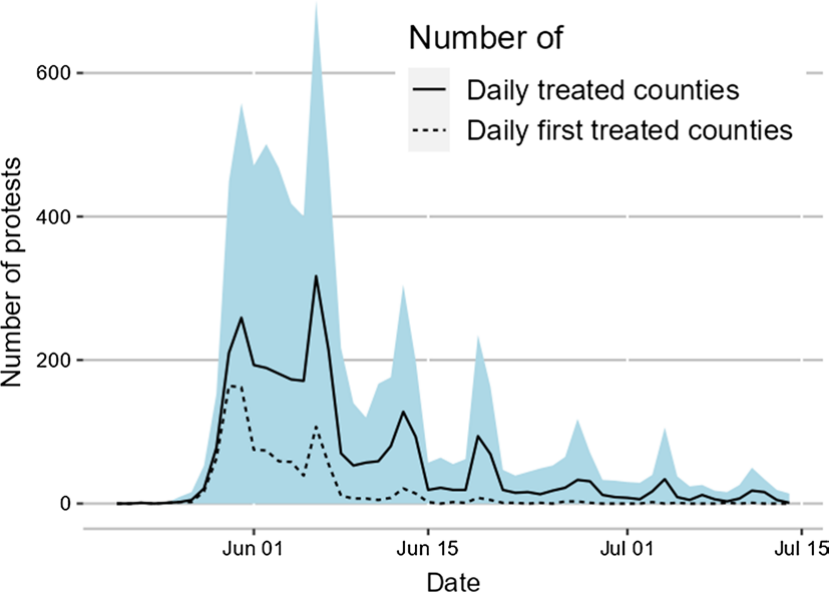
Number of daily BLM protests in the United States. *Notes:* The blue shaded area shows the raw number of events as recorded in the data. The solid line shows the number of counties treated per day. The dashed line shows the number of counties treated for the first time. The treatment period starts on May 26 and ends on June 15. (Color figure online)

The BLM protests started following the death of George Floyd in police custody on May 25 and quickly spread throughout the United States. Figure 2 shows the total number of distinct protests per day in the area shaded in light blue. The number of distinct protests peaked on the first weekend of June and remained high for around a week, with over 400 recorded protests every day. This number then declined sharply and fell to only a few dozen protests per day at the end of June. Since many of these protests involved only a handful of individuals, we restrict our sample to “large-scale” protests, which we define as a protest with at least 100 participants or one classified as non-peaceful.^8^ The solid line in Fig. 2 reflects the same pattern, though the number of such protests was only around 200 per day during the peak.

Due to the concentration of events around the death of George Floyd, we define exposure to BLM protests for each county in terms of having experienced a large-scale BLM protest in that county between May 26 and June 15. These counties are considered treated in all subsequent periods. This design choice is motivated by the dashed line in Fig. 2, which shows the number of counties experiencing their first large-scale protest on a given day. The vast majority of large-scale protests after June 15 occurred in counties that had previously experienced large-scale protests, with only a negligible number of counties experiencing their first large-scale protest after this date. Throughout, we omit these latter counties from our analyses to prevent them from biasing our results.

### Additional data

We further supplement our dataset with data on county-level demographics and past election results from the US General Elections 2018—Analysis Dataset, made available through the MIT Elections Lab.^9^ Specifically, from this dataset we collect county-level information on presidential vote shares in the 2016 election, as well as information on total population, percentage of non-white population, and median household income.

## Empirical strategy

Our empirical strategy relies on the comparison of voter registrations over time across counties with and without major BLM protests in a two-way fixed-effects framework. In addition, we match counties one-to-one based on predetermined variables to create a control group that closely resembles the treatment group with regard to demographics, political preferences, and voter registration dynamics. We define counties that experienced at least one large-scale BLM-related protest before June 15 as being exposed while the remaining counties are considered unexposed. We define the first observation following the first recorded large-scale BLM protest in the county as the onset of the treatment period, with these counties remaining in the treatment group in all post-treatment periods. As discussed above, we choose this operationalization over a staggered treatment design because the vast majority of counties that ever experienced BLM-related protests did so before June 15 and were subsequently subject to recurring bouts of protests.

The empirical model we employ is as follows:

1 $$Y_{{{\text{c}},{\text{s}},t}} = \alpha_{{\text{c}}} + \beta_{{{\text{s}},t}} + \mathop \sum \limits_{t} D_{{{\text{c}},{\text{s}},t}} \delta_{t} + \varepsilon_{{{\text{c}},{\text{s}},t}}$$

where $Y_{{{\text{c}},{\text{s}},t}}$ denotes the number of voter registrations scaled by county population. $\alpha_{{\text{c}}}$ and $\beta_{{{\text{s}},t}}$ indicate county fixed effects and state-by-period fixed effects, respectively. Further, $D_{{{\text{c}},{\text{s}},t}}$ denotes a set of indicator variables that take a value of 1 for the observation in period *t* and county *c* if the county experienced a large-scale BLM protest between May 26 and June 15. The coefficients $\delta_{t}$ therefore estimate the difference in per-capita voter registrations between treated and untreated counties in each time period. We omit $D_{{{\text{c}},{\text{s}},t}}$ for the last pre-treatment period so that the remaining coefficients can be interpreted relative to this base period. Throughout, we cluster standard errors at the county level.

The fixed effects alleviate several potential threats to identification. First, county fixed effects account for persistent differences across counties that might be correlated with both the presence of BLM protests and voter registrations, such as size, demographic makeup, and unobserved factors like the costs of mobilizing voters. The state-by-period fixed effects further control for state-specific policies that are common to all counties within the state. Importantly, this method accounts for statewide responses to the COVID-19 pandemic and state-specific idiosyncrasies in the reporting and administration of voter registrations.^10^

Despite these detailed controls, applying our estimation strategy to the full sample is unlikely to result in valid inferences, since counties are highly heterogeneous in population and the probability of having a protest increases with the county’s population. We consider it unlikely that highly populous counties are subject to the same trends in voter registration as less populous ones; hence we instead pursue a two-step strategy similar to that of Jäger and Heining (2019) by employing coarsened exact matching (CEM;Ho et al., 2011; Iacus et al., 2012) to create a balanced sample on the basis of predetermined characteristics prior to conducting the two-way fixed-effects analysis.^11^ Importantly, since matching is not performed on prior trends in voter registrations, the forward-looking coefficients ahead of the protests remain informative about the plausibility of the common trends assumption.

In our main specification, we match counties based only on population. Specifically, we transform this continuous variable into 100 strata. The matching mechanism omits all counties within strata in which there exist no observations of opposite treatment status. The remaining observations in each stratum are then subjected to Mahalanobis matching so as to achieve a one-to-one match between treated and untreated observations. Throughout the article, we present extensive robustness checks, varying the number of strata, including additional variables, and using weighted regressions instead of one-to-one matching to ensure that the specific operationalization of the matching procedure is not driving our results.^12^

Table 1 shows (unweighted) summary statistics of the treated and untreated counties in the full sample and in the matched sample. It shows that in the full sample, treated and untreated counties differ substantially on most predetermined variables, with large-scale BLM protests being more frequent in more populous counties with a more Democratic-leaning population. This result is due to large urban counties, virtually all of which experienced BLM protests during our sample period. The matching procedure retains only those treated counties for which a comparable untreated observation is available. This process removes many of the counties at the tail end of the population size distribution, thereby aligning the two groups more closely with respect to their predetermined characteristics, as displayed in the bottom panel of Table 1. In additional robustness checks, we match counties on a larger set of predetermined variables to achieve an even more balanced sample, but for transparency we rely on the most parsimonious matching strategy for our main results.

**Table 1:** Summary statistics

	All counties			
	With protest (657)		Without protest (1478)	
% Registered	67.4	(0.1)	66.0	(0.1)
% Trump vote 2016	54.6	(0.1)	67.9	(0.1)
Population	238.2	(432.1)	31.8	(58.2)
% White	75.3	(18.4)	78.2	(20.0)
Median income	52.2	(13.5)	45.4	(10.8)

	Matched counties			
	With protest (412)		Without protest (412)	
% Registered	67.3	(0.1)	66.0	(0.1)
% Trump vote 2016	58.4	(0.1)	63.8	(0.1)
Population	77.1	(95.5)	75.8	(95.8)
% White	80.0	(16.2)	77.3	(18.0)
Median income	48.5	(10.9)	48.8	(11.9)

Mean and standard deviation (in parentheses) of predetermined county characteristics. All counties are equally weighted. The top panel shows the summary statistics for the full sample; the bottom panel shows the sample as matched based on population. The number in parentheses in the header indicates the number of counties that constitute this subsample. Population is measured in thousands of inhabitants and median income is measured in thousands of dollars

## Results

We begin with a discussion of our main results, before investigating their robustness and further decomposing the findings by party affiliation and characteristics of the protests—in particular, whether they were associated with violent behaviors.

**Fig. 3:**
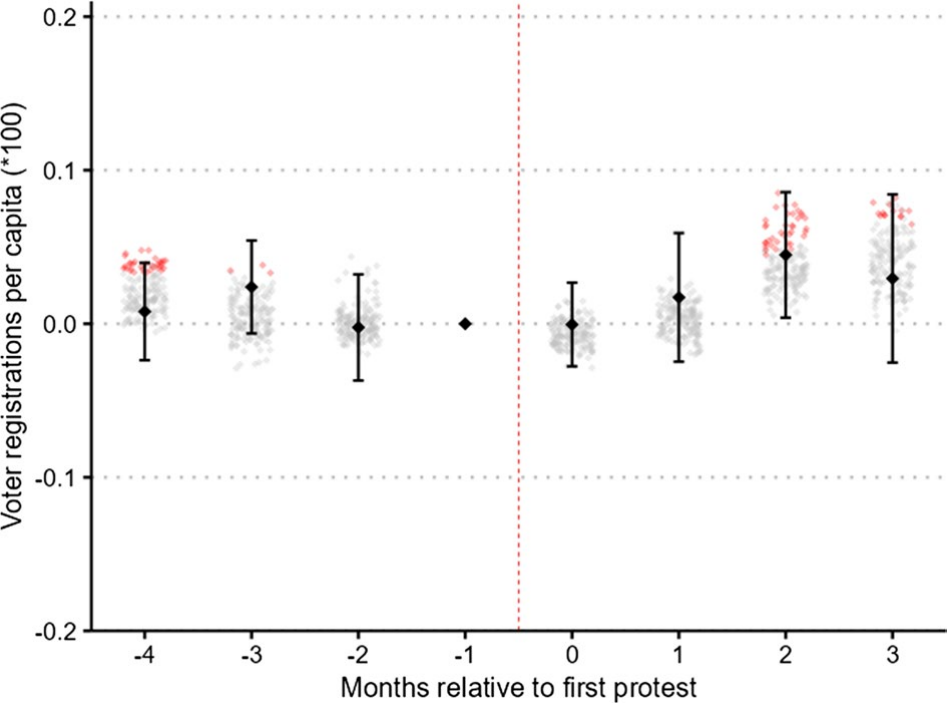
Event-study coefficients: total voter registrations. *Notes:* Event-time signifies months relative to the first recorded large-scale BLM protest between May 26 and June 15. The black diamonds are the estimated coefficients from an OLS regression of Eq. (1), with counties being matched one-to-one based on 100 population strata. Error bars are 95% confidence intervals with standard errors clustered at the county level. Small diamonds in the background indicate estimated coefficients from alternative specifications with different matching strata, matching on additional variables, and weighted M:1 matching instead of 1:1 matching. Red color indicates statistical significance at the 5% level. (Color figure online)

Figure 3 presents our main results. The black diamonds represent coefficient estimates obtained from an ordinary least squares (OLS) regression of Eq. (1) with voter registrations per capita as the dependent variable. Counties are matched one-to-one based on population. In an attempt to account for the possible sensitivity of our results to the matching procedure, around each main coefficient we plot the estimated coefficients from possible alternative operationalizations. Specifically, we present coefficients from 83 alternative specifications, varying the set of variables included in the matching procedure, the number of matching strata, and the weighting scheme. To enable some basic inferences, we indicate in red all the estimated coefficients that reached statistical significance at the 5% level.

In our main specification, after applying the matching algorithm, we are left with 6368 observations from 796 counties. The coefficients are precisely estimated and give little indication of diverging trends prior to being treated. Moreover, we fail to reject the null hypothesis of no effect immediately following the protests. Although the estimated coefficient becomes positive and statistically significant at the 5% level in the second post-treatment period, we caution against interpreting this finding as evidence of positive mobilization effects, since the estimate is not robust to alternative choices of matching parameters. Further, even if taken at face value, the estimated point coefficient 2 months after the protests corresponds to an increase in per-capita voter registrations of only 0.0005.^13^

The corresponding time-invariant estimate is presented in column 1 of Table 2. In line with our interpretation of the time-varying coefficients, the overall effect is estimated to be positive, but small in magnitude and not statistically significant. The point coefficient, if taken at face value, suggests that local BLM protests resulted in a per-period increase in per-capita voter registrations of 0.00015. For the median county in our estimation sample with around 28,500 registered voters in February 2020, this corresponds to a monthly increase of four voters.

**Table 2:** Main results

	Voter registrations per capita (× 100)			
	(1)	(2)	(3)	(4)
Protest	0.01537	−0.00867	0.00950	
	(0.01369)	(0.00785)	(0.01082)	
Violent protest				−0.00211
				(0.02706)

Party	Total	Democratic	Republican	Total
State-month fixed effects	Yes	Yes	Yes	Yes
County fixed effects	Yes	Yes	Yes	Yes
Observations	6368	3632	3632	1568

Standard errors clustered on the county level.

*Indicates significance at the 5% level. In a first stage, counties are matched one-to-one using coarsened exact matching on 100 population strata. Column 4 compares counties with peaceful BLM protests to counties with non-peaceful BLM protests

The significance indication * is standard, but our results are insignificant

### Robustness

**Fig. 4:**
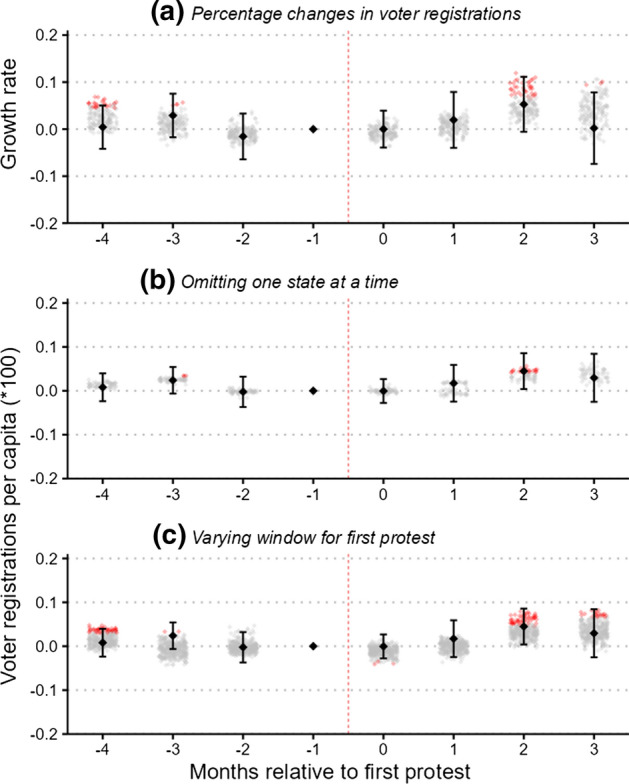
Robustness. *Notes:* Event-time is months relative to the first recorded large-scale BLM protest between May 26 and June 15. (**a**) Equivalent to Fig. 3 but uses as the dependent variable the growth rate of voter registrations. The small diamonds in (**b)** are estimated coefficients with one state omitted at a time; the main specification is reproduced in black. (**c)** Versions of Fig. 3, varying the cutoff date before which a protest has to occur for it to be considered as treated. Specifically, it combines four versions of Fig. 3, with the protest date cutoff varying in increments of 5 days from June 10 to June 25. In panel **c**, the definition of event-time might therefore differ across specifications

There are a few potential concerns related to the validity of our findings. First, as described in the data section, states differ widely not only with respect to the rules surrounding voter registration, but also regarding the communication of voter registration statistics to the public. Although we attempt to reduce the influence of such state-specific idiosyncrasies through the inclusion of high-dimensional fixed effects, one might nonetheless be concerned that any single state might exert undue influence on our estimated coefficients. Panel (b) of Fig. 4 presents event-study coefficients, where every gray diamond represents a separate regression in which one state is removed from the data. As a visual aid, we overlay the coefficient and confidence interval from the main specification in black. Overall, there appears to be relatively little variation in the estimated coefficients. This insight is also relevant in view of our incomplete coverage of states, as it suggests that the estimated effects are not driven by any specific state and thus should be generalizable to other states not included in our sample.

Second, we test whether our findings are robust to alternative operationalizations of the outcome variable. Specifically, in panel (a) of Fig. 4, we present event-study results while using as the dependent variable the growth rate of monthly voter registrations (i.e., voter registrations divided by total registrations in the preceding period). While the coefficients are not directly comparable, the results are qualitatively similar.

Finally, we analyze the role of two important design choices in our definition of large-scale protests that might influence our estimates. In all analyses presented thus far, we defined a county as treated if it experienced a protest with at least 100 participants before June 15. As discussed in Sect. 3, a small number of counties experienced their first large-scale protest after this date and are coded as untreated in our analysis. Although removing these later-treated counties from the sample prevents them from influencing the estimates, we nonetheless investigate whether the findings are sensitive with respect to the specific date chosen as the cutoff. Specifically, in panel (c), we repeat our analyses for date cutoffs ranging from June 10 to June 25 in increments of 5 days.^14^ Overall, the figure suggests that varying the date does not change our estimated coefficients in a meaningful way.

**Fig. 5:**
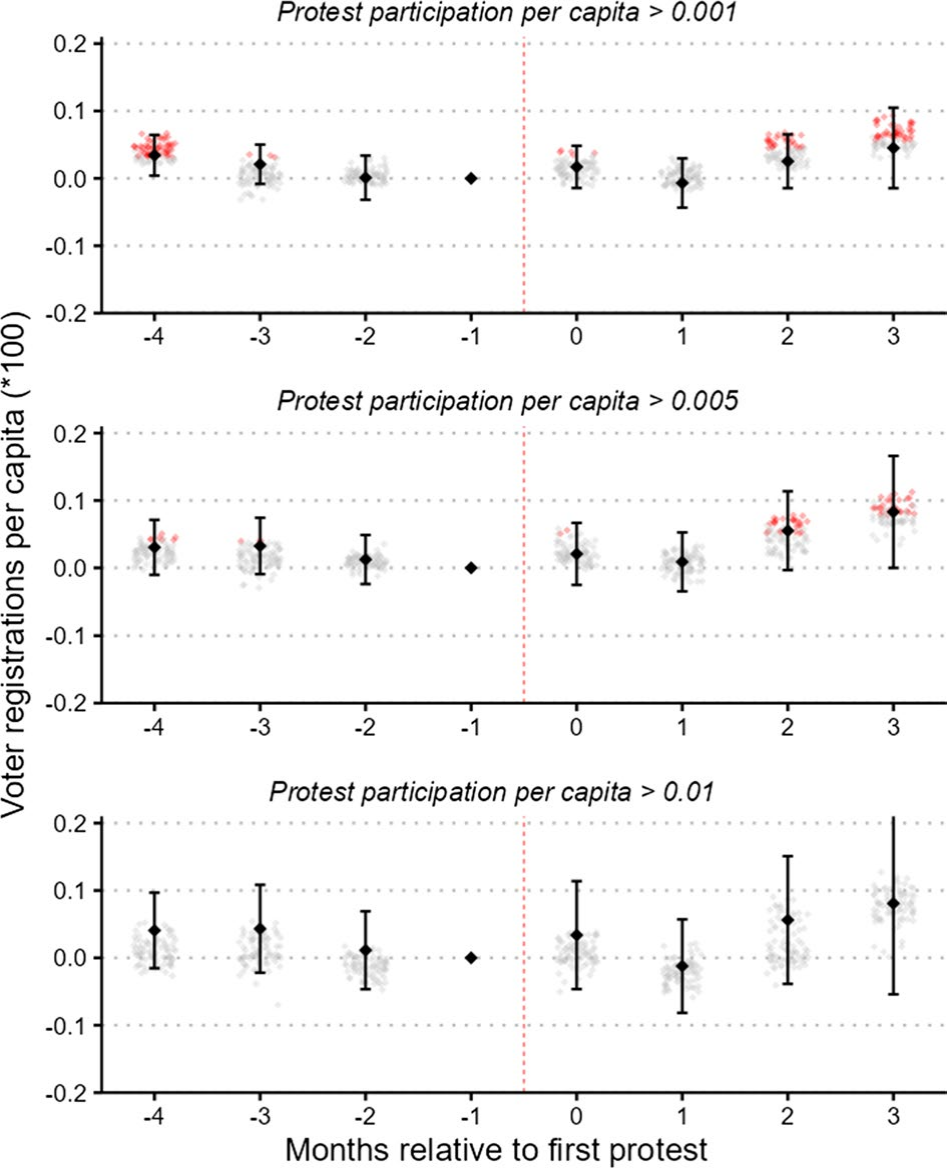
Robustness: relative protest size. *Notes:* Event-time is months relative to the first recorded large-scale BLM protest between May 26 and June 15. The panels are equivalent to Fig. 3 but employ different size cutoffs above which protests are considered “large-scale”

Furthermore, since our definition of a “large-scale” protest is based on participation in absolute terms, it ignores the fact that in a small county, even a protest with a few dozen participants might be meaningful, whereas 100 participants can hardly be considered a large number in a county of several million inhabitants. To address this issue, in Fig. 5 we implement an alternative definition of the size cutoff based on relative size. Specifically, we vary the size cutoff between 0.1% (top panel) and 1% (bottom panel) of the county population. Unlike in the main analysis, where violent protests were considered “large-scale” irrespective of their size, here we base inclusion in the sample exclusively on protest size. We find that the estimates produced from these specifications are similar to those generated by the 100-participant cutoff, although increasing the threshold reduces the effective number of observations and thereby renders the estimates less precise.

### Heterogeneity

Thus far, we have limited our analysis to aggregate mobilization effects. However, these aggregate effects might mask important heterogeneity, with protests mobilizing only some voters but not others. Specifically, mobilization effects might depend on whether potential voters perceived the protests as legitimate criticisms of social injustices or as violent riots. Based on this rationale, we explore heterogeneity along two dimensions: first, we test for differential mobilization effects across party lines, since the narratives used to describe the protests differed across the political spectrum, with conservative news outlets more frequently relating the BLM protests to riots or looting (FiveThirtyEight, 2020). Second, we test whether mobilization effects depend on the degree of violence associated with the protests.

**Fig. 6:**
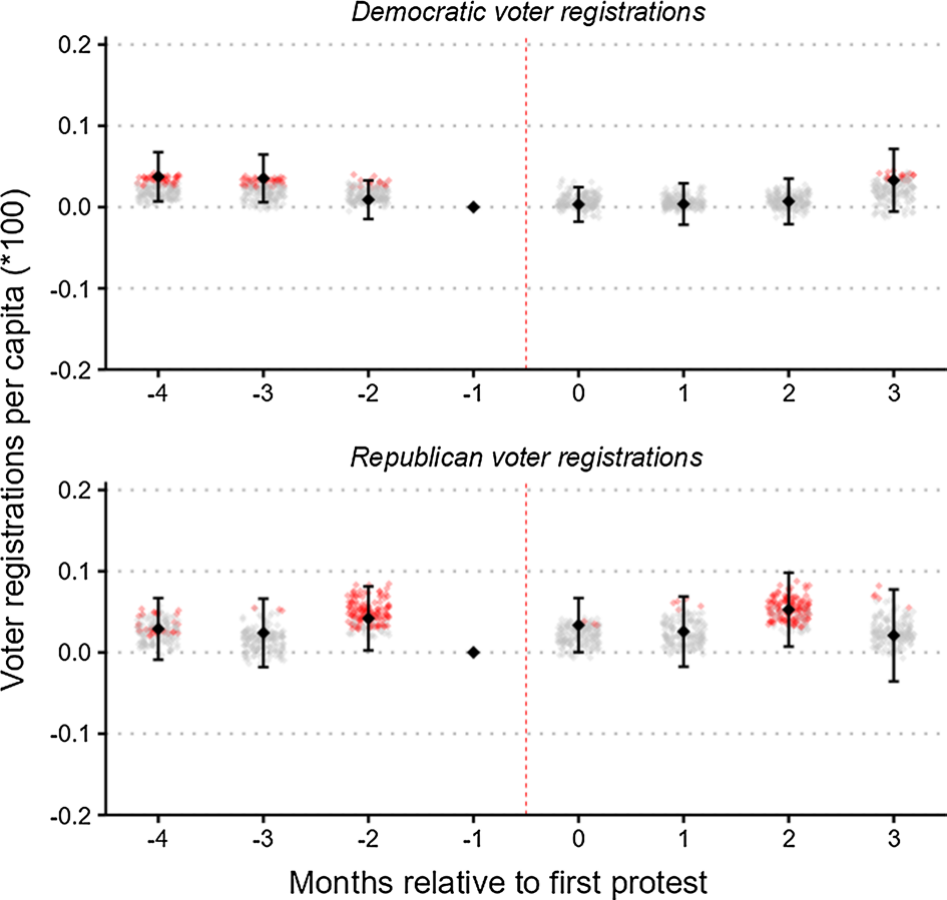
Heterogeneity by party affiliation. *Notes:* Event-time is months relative to the first recorded large-scale BLM protest between May 26 and June 15. The black diamonds are the estimated coefficients from an OLS regression of Eq. (1), with counties being matched one-to-one based on 100 population strata. Error bars are 95% confidence intervals with standard errors clustered at the county level. Small diamonds in the background indicate estimated coefficients from alternative specifications with different matching strata, matching on additional variables, and weighted M:1 matching instead of 1:1 matching. Red diamonds are statistically significant at the 5% level. (Color figure online)

Figure 6 presents event-study estimates using as the dependent variable per-capita voter registrations, with the affiliation listed as Democrat in panel (a) and as Republican in panel (b). Because not all states report party affiliation in their voter registration data, this step cuts our sample by almost half, to 3,632 observations from 454 counties. Although this reduction in sample size reduces the precision of our estimates, this loss of precision appears to be largely compensated for by a reduction in the variation of the outcome variable.

We are unable to reject the null hypothesis of no change in voter registrations for both Democratic and Republican voters in all periods. We should note, however, that due to the reduced sample size, our matching strategy is somewhat less effective in aligning parallel trends, although there still appears to be no clear pattern in voter registration patterns following the protests. The corresponding time-invariant estimates are presented in columns 2 and 3 of Table 2 and are similarly insignificant. The 95% confidence intervals of the time-invariant estimates are [−0.00024, 0.00007] and [−0.00012, 0.00031] for Democrats and Republicans, respectively. Hence, our estimates allow us to rule out meaningful reductions in voter registrations for both major parties. Further, our estimates also provide evidence against large positive adjustments, since even the upper limits of the 95% confidence intervals still constitute small effect sizes.

As a final test, we investigate the heterogeneity of treatment effects across peaceful and non-peaceful protests. In the reporting on the BLM protests, their characterization as violent has been an influential narrative, although most protests in our data are classified as peaceful, with only a small subset of counties ever experiencing non-peaceful protests.

Since we are interested in testing whether peaceful and non-peaceful protests differ in their ability to mobilize voters, we restrict the sample to counties that experienced a large-scale protest and estimate the effect of violent protests within this sub-sample. As further explained in the appendix, we combine all events not labeled as a “peaceful protest” by the ACLED into a binary treatment variable for a “non-peaceful event.” This means that we estimate an average effect across all non-peaceful events, irrespective of who initiated the violence or the overall extent and shape of the violent interactions.

**Fig. 7:**
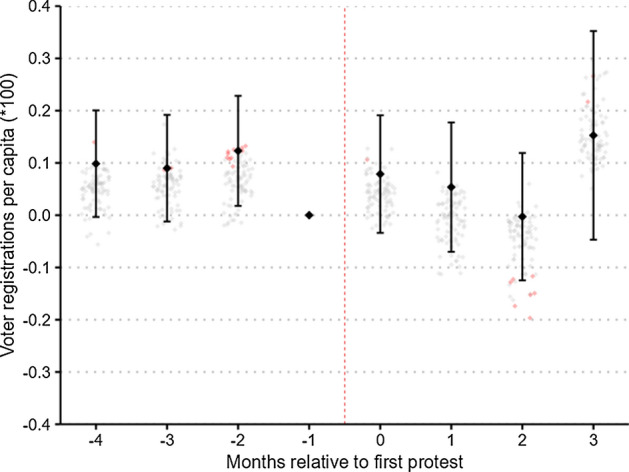
Effect of non-peaceful protests. *Notes:* Event-time is months relative to the first recorded non-peaceful BLM protest between May 26 and June 15. The figure is equivalent to Fig. 3, except that counties are considered as treated only if they experienced a non-peaceful protest between May 26 and June 15. Matching is performed within counties that experienced a large-scale BLM protest, thereby comparing counties with peaceful protests to those experiencing non-peaceful protests

Figure 7 presents the estimated time-varying coefficients and column 4 of Table 2 presents time-invariant results. As in previous analyses, all estimated treatment effects are statistically insignificant. However, because few of the protests in our sample were classified as violent, the significant reduction in sample size results in a drastic loss of precision, with confidence intervals increasing by a factor of roughly 4. While our estimates are still reasonably precise, this implies that we cannot rule out potentially meaningful effect sizes. The confidence interval of the time-variant coefficient has an upper bound of 0.0005, or around 14 voters in the median county. In very large counties, however, monthly increases of this magnitude could be considered a success from a mobilization perspective.

Overall, the heterogeneity analyses presented above give little indication of meaningful mobilization effects of local protests on potential voters of either party and irrespective of whether the protests were associated with violence.

## Discussion and conclusion

In this study, we provide new insights into the effects of political protests on political participation, using voter registrations as a high-frequency measure of participation. In contrast to previous studies on the mobilization effects of political protests, we find little evidence that local BLM protests significantly increased overall political participation, across a wide range of specifications. We further show that there is little evidence that this aggregate null effect masks heterogeneity across party lines or across peaceful and non-peaceful protests.

There are several possible explanations of why our estimated effects differ from those of previous studies. First, our study uses voter registrations as its main measure of political engagement. This choice of dependent variable is tightly linked to our identification strategy, which aims to exploit timing variation to identify the effects of protests and therefore requires data available with high frequency, which is obviously unattainable for voter turnout.^15^ As a consequence, we estimate the mobilization effect in a sub-population that has previously been politically inactive. While mobilization of this demographic has been an important goal in recent years, its non-representative nature limits the generalizability of our findings to more traditional measures of political participation such as voter turnout. For example, the protests might have mobilized politically interested citizens who were already registered to vote, which would not be captured by our analysis.

A second possible explanation lies in the vast scale and media coverage of the BLM movement and the associated protests. As our estimation strategy is based on the comparison of different US counties over time, it can identify only the local effects of protests beyond an aggregate effect common to all counties. Thus, the null results might be a consequence of the widespread coverage that increased awareness of the issues emphasized by BLM even in areas not directly exposed to protests.

**Fig. 8:**
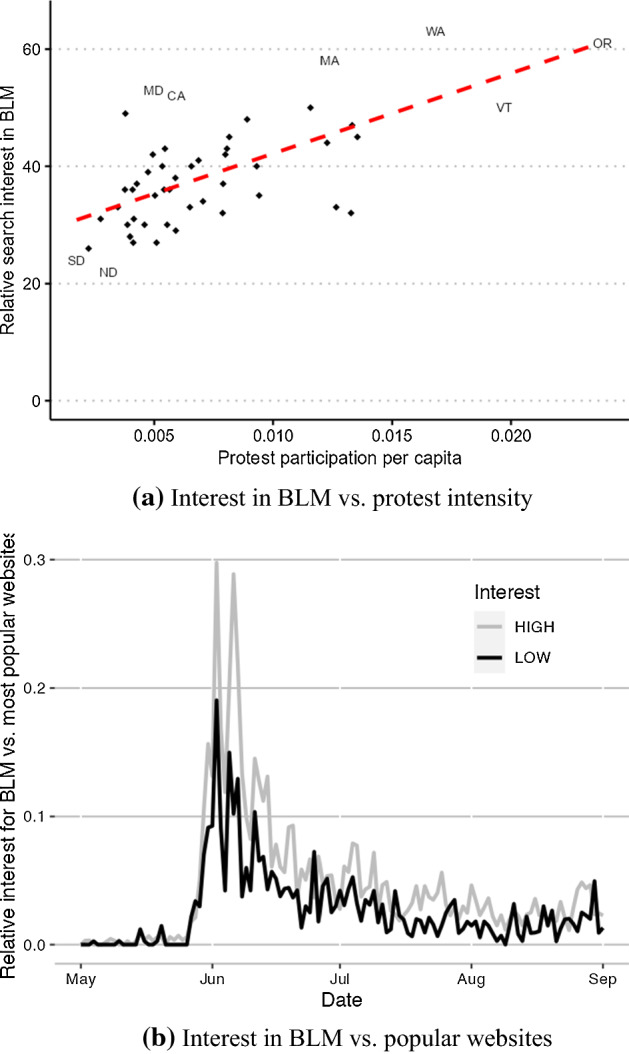
Relative interest in BLM as measured by Google searches. *Notes:* (**a)** Correlation between the overall interest in the topic “Black Lives Matter” on Google between May 20 and June 14, 2020, and protest participation per capita in the same time period. The measure of protest participation omits protests for which no size was reported in our data. (**b)** Relative search volume for the term “Black Lives Matter” compared to the average search volume of the four most popular websites—Google, Amazon, YouTube and Facebook—for the five states with the highest and lowest relative search interest. High-interest states are Massachusetts, Maryland, Oregon, California, and Washington; low-interest states are North Dakota, South Dakota, Alabama, West Virginia, and Louisiana

While an in-depth investigation of the role of this factor would require county-level data on news coverage that is not presently available to us, we explore this hypothesis using state-level data on Google searches retrieved from Google Trends. Panel (a) of Fig. 8 presents a simple scatter plot of the relative search interest in the topic “Black Lives Matter” during the period from May 20 to June 14, across states against the number of BLM protesters per capita identified in our data. The figure suggests that, as one might expect, there is a strong positive correlation between protest participation and the search intensity for terms related to BLM. However, in line with the hypothesis that news coverage and social media popularized BLM beyond the areas subject to protests, even the states with the lowest protest participation still exhibited around one-half to one-third of the search interest of the highest-interest states.

Panel (b) of Fig. 8 provides further evidence in favor of this view. Since panel (a) displays only the relative search intensity across states, it does not allow us to draw any conclusion about overall search volumes. Therefore, in panel (b), we plot a time series of the search intensity for the topic “Black Lives Matter” relative to the average search intensity for the four most popular websites in the United States, separately for the states with the highest and lowest search interest as identified in panel (a). In the high-interest states, at its peak, the BLM topic received around one-third of the number of searches of the most popular websites. Importantly, the peak search interest in the low-interest states corresponded to 15% of the search intensity for the most popular websites, which still constitutes considerable interest.

Overall, the data indicate that interest in the BLM movement was high across all states, even those with relatively little direct exposure to protests. Although this analysis falls short of a thorough mediation analysis, it nonetheless suggests the possibility that widespread news coverage and access to social media might have weakened the need for direct exposure to protests to mobilize voters.

## Figures and Tables

**Fig. 9:**
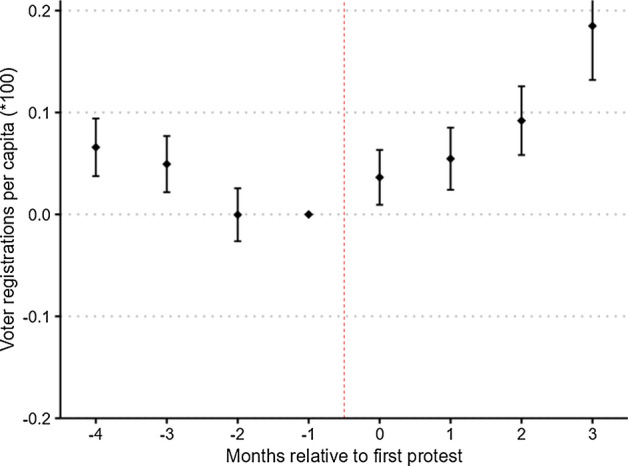
Regression coefficients without matching

**Fig. 10:**
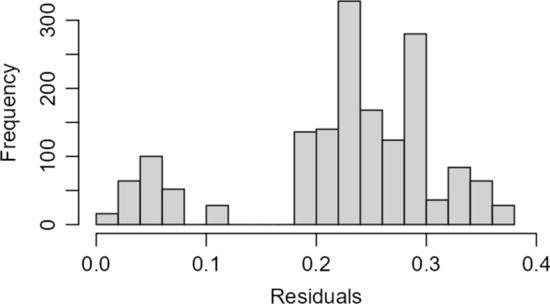
Distribution of residuals

## Appendix 1

### Voter data: sample descriptives

See Table 3.

**Table 3:** Sample descriptives

Sample	*N*	% Trump 16	Mean pop	Total pop	Income	% rural	% white
No	976	62.1	117,077	114,266,978	48,465	59.8	77.5
Yes	2135	63.8	95,335	203,539,975	47,522	57.9	77.3

Descriptive statistics of counties in our sample of voter registration data compared with counties not in the sample. The third column shows the average county-level vote share for Donald Trump in the 2016 election. Income in US dollars

### Protest data: variable definitions

Each event-day is a unit of observation and contains information on the event date, the location, the type of event (“riot” or “protest”), the sub-type of the event, and the groups and organizations involved. The information on these events comes from media outlets, such as newspapers and websites. The events are coded manually by researchers and reviewed twice by different researchers (Armed Conflict Location and Event Data Project [ACLED], 2019). Furthermore, each event contains a note that provides additional information on the size and nature of the event. We use the information about event size contained in the notes in the ACLED dataset. When no information on the size of the protest is available, we infer whether the protest was significant from the event description. We count a protest as significant if a known organization organized the protest, if the events described could have happened only with a certain degree of anonymity (e.g., looting, attacks on law enforcement), or if law enforcement had to resort to crowd control tactics such as tear gas or rubber bullets. Peaceful protests without size estimates and without any other notable characteristics were classified as “insignificant” from the perspective of public attention.

We filter for all events labeled as either “protests” or “riots.” We label an event as a “Black Lives Matter” event if the list of main actors includes BLM activists. Since the ACLED data contain protests of all sizes, including very small gatherings, we impose a lower cutoff of 100 participants to limit our analysis to events that attracted public attention.

Events are further categorized as “peaceful protests,” “mob violence,” “violent demonstrations,” “protests with intervention,” or “excessive force against protesters.” For the heterogeneity analysis, where we estimate the effect of experiencing a non-peaceful event, we consider all counties that experienced an event that was not a “peaceful protest” during the treatment period to be treated.

### Additional results

Figure 9 shows the event-study coefficients of Eq. (1) without matching counties based on population. The point coefficients post-treatment are larger than in the matched sample and are statistically significant. This finding is likely driven by the facts that larger urban areas had especially strong initiatives to register voters and that they also all experienced large protests. The anticipatory effects reflect the fact that these counties were already registering more voters prior to the George Floyd protests.

### Two-way fixed effects

Recently, a number of authors have pointed out that two-way fixed-effects models have the potential to produce negative weights, which, in the presence of heterogeneous treatment effects, can result in the estimated coefficients being of the opposite sign of all treatment effects. Although De Chaisemartin and d’Haultfoeuille (2020) note that this problem is more likely to arise in staggered designs when a large number of groups are treated in a specific period or groups have been treated for a long period, we nonetheless explore whether this issue is relevant in our setting. For this purpose, we first estimate a regression of treatment status on the set of county fixed effects and state-month fixed effects using our main estimation sample. We then calculate the residuals from this regression for all treated observations. As shown by De Chaisemartin and d’Haultfoeuille (2020), the weights applied by the two-way fixed-effects model are a rescaled version of these residuals. As a result, the sign of these residuals informs us about the sign of the weights.

Figure 10 shows the distribution of these residuals. None of the residuals are negative, indicating that no group-level treatment effects are weighted negatively.

## Footnotes

1 See Kurrild-Klitgaard (2004) for a more extensive overview of the literature that aims to rationalize protest participation.

2 A few studies have analyzed the BLM movement in other contexts, such as the demand for National Football League games (Brown & Sheridan, 2020) or lethal use of force by the police (Campbell, 2021).

3 FiveThirtyEight (2020) reports that Fox News mentioned looting or rioting almost twice as much as other cable networks.

4 For example, previous research has shown that disastrous events affect the behavior of people in the same television market (Gallagher, 2014), suggesting a reduced role of local effects.

5 The sample contains these 32 states: Alabama, Arkansas, Colorado, Delaware, Florida, Idaho, Illinois, Iowa, Kentucky, Louisiana, Maine, Missouri, Nebraska, Nevada, New Jersey, New Mexico, New York, North Carolina, Ohio, Oklahoma, Oregon, Pennsylvania, Rhode Island, South Dakota, Texas, Vermont, Virginia, Washington, West Virginia, Wisconsin, Wyoming.

6 However, even in states where voters can register with a party, the incentives to do so differ across states. For example, in many states voters need to be registered with a specific party to vote in the relevant primary election, whereas others, such as South Dakota, allow unaffiliated voters to participate in the Democratic primary.

7 We show in the appendix that counties in the sample are, overall, comparable to excluded counties in terms of average population, the vote share for Donald Trump in 2016, median household income, and the share of rural and white inhabitants.

8 We provide robustness checks with different size cutoffs based on the number of protesters relative to county population.

9 The dataset can be downloaded from https://github.com/MEDSL/2018-elections-unoffical/blob/master/election-context-2018.md and contains information that was compiled from the US Department of Agriculture, various IPUMS (Integrated Public Use Microdata Series) databases and Pettigrew (2016).

10 Recent studies have raised concerns about negative weighting in regressions with two-way fixed effects (De Chaisemartin and d’Haultfoeuille 2020). We discuss the relevance of this literature for our study in the appendix and present a diagnostic test to show that our analysis is not plagued by negative weights.

11 The results of a specification without matching are available in the appendix. Although the post-treatment effects are highly significant, it is evident that without the matching procedure, counties with protests were already registering significantly more voters prior to the George Floyd protests. Hence, we conclude that in the full sample, the parallel trends assumption is likely to be violated.

12 In what follows, weighted regressions always refer to a regression setup in which the Mahalanobis matching step is omitted; instead, all observations in strata for which observations of the opposite treatment status exist are retained and weighted to achieve balance.

13 This corresponds to around 0.1 standard deviations, an effect size usually classified as small or very small.

14 For dates later than June 15, our sample is no longer balanced across all event-time periods, since we are missing data for Texas in the third post-treatment period.

15 Previous studies of the causal effects of protests have instead resorted to instrumental variables strategies, such as using the variation in rainfall across locations as an instrument for the presence or size of protests (Collins & Margo, 2007; Madestam et al., 2013). Implementing this strategy in our setting is not feasible, as protests were spread out across several weeks. This renders rainfall a poor predictor of protest presence, resulting in a weak first stage.
